# Bcl-2-like protein 13 is a mammalian Atg32 homologue that mediates mitophagy and mitochondrial fragmentation

**DOI:** 10.1038/ncomms8527

**Published:** 2015-07-06

**Authors:** Tomokazu Murakawa, Osamu Yamaguchi, Ayako Hashimoto, Shungo Hikoso, Toshihiro Takeda, Takafumi Oka, Hiroki Yasui, Hiromichi Ueda, Yasuhiro Akazawa, Hiroyuki Nakayama, Manabu Taneike, Tomofumi Misaka, Shigemiki Omiya, Ajay M. Shah, Akitsugu Yamamoto, Kazuhiko Nishida, Yoshinori Ohsumi, Koji Okamoto, Yasushi Sakata, Kinya Otsu

**Affiliations:** 1 Department of Cardiovascular Medicine, Graduate School of Medicine, Osaka University, 2-2 Yamadaoka, Suita, Osaka 565-0871, Japan; 2 Laboratory of Mitochondrial Dynamics, Graduate School of Frontier Biosciences, Osaka University, 1-3 Yamadaoka, Suita, Osaka 565-0871, Japan; 3 Laboratory of Clinical Science and Biomedicine, Graduate School of Pharmaceutical Sciences, Osaka University, 1-6 Yamadaoka, Suita, Osaka 565-0871, Japan; 4 Cardiovascular Division, King's College London British Heart Foundation Centre of Excellence, 125 Coldharbour Lane, London SE5 9NU, UK; 5 Faculty of Bioscience, Nagahama Institute of Bio-Science and Technology, 1266 Tamura-Cho, Nagahama, Shiga 526-0829, Japan; 6 Frontier Research Center, Tokyo Institute of Technology, 4259-S2-12 Nagatsuta-cho, Midori-Ku, Yokohama, Kanagawa 226-8503, Japan

## Abstract

Damaged mitochondria are removed by mitophagy. Although Atg32 is essential for mitophagy in yeast, no Atg32 homologue has been identified in mammalian cells. Here, we show that Bcl-2-like protein 13 (Bcl2-L-13) induces mitochondrial fragmentation and mitophagy in mammalian cells. First, we hypothesized that unidentified mammalian mitophagy receptors would share molecular features of Atg32. By screening the public protein database for Atg32 homologues, we identify Bcl2-L-13. Bcl2-L-13 binds to LC3 through the WXXI motif and induces mitochondrial fragmentation and mitophagy in HEK293 cells. In Bcl2-L-13, the BH domains are important for the fragmentation, while the WXXI motif facilitates mitophagy. Bcl2-L-13 induces mitochondrial fragmentation in the absence of Drp1, while it induces mitophagy in Parkin-deficient cells. Knockdown of Bcl2-L-13 attenuates mitochondrial damage-induced fragmentation and mitophagy. Bcl2-L-13 induces mitophagy in Atg32-deficient yeast cells. Induction and/or phosphorylation of Bcl2-L-13 may regulate its activity. Our findings offer insights into mitochondrial quality control in mammalian cells.

## Abstract (web summary)

Atg32 is required for mitophagy in yeast, however, a mammalian homologue of this protein has not been identified. Murakawa *et al*. identify Bcl-2-like protein 13 as a functional homologue of Atg32 in mammalian cells, and show that this protein can rescue mitophagy in Atg32-deficient yeast cells.

## Graphical abstract

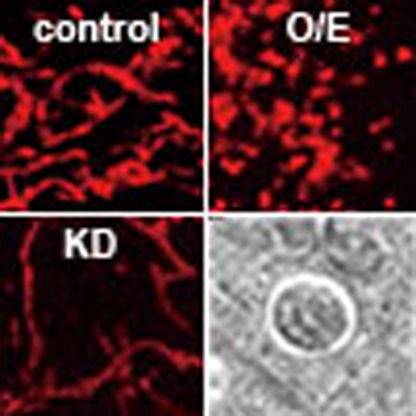

Mitochondria are subcellular organelles that produce energy through oxidative phosphorylation. Dysregulated mitochondrial activity results in generation of reactive oxygen species as a by-product of oxidative phosphorylation, which cause damage to DNA and proteins^1^. Thus, mitochondrial quality control is essential for normal cellular functions. Macroautophagy (hereafter referred to autophagy) is responsible for mitochondrial quality control^1^. There are two types of autophagy, non-selective and selective autophagy. Non-selective autophagy sequesters bulk cytoplasm and organelles engulfed by isolation membrane as cargos to autophagosomes^2^. These then undergo fusion with lysosomes, allowing degradation of the cargo. In contrast, selective autophagy targets specific proteins or organelles as cargos, such as mitochondria and peroxisomes. The degradation of damaged mitochondria is mediated by a selective type of autophagy, mitophagy^3^. Dysregulation of mitophagy is implicated in the development of neurodegenerative diseases, such as Alzheimer’s disease and Parkinson’s disease as well as metabolic diseases, heart failure and ageing^3^.

Mitochondrial morphologies change continuously through actions of fission and fusion (collectively termed mitochondrial dynamics). In yeast^4^ and mammalian cells^5^, mitophagy is reported to be preceded by mitochondrial fission, which divides elongated mitochondria into pieces of manageable size for engulfment by isolation membrane. To date, more than 30 autophagy-related (Atg) genes have been identified, which function as molecular machinery for autophagy^2^. In yeast, Atg32 is essential for mitophagy and functions as a receptor of mitophagy through its interaction with Atg8 and Atg11 (ref. 6, 7). It has a single transmembrane domain in the C-terminal fifth of the protein, spanning outer mitochondrial membrane (OMM) and contains a WXXI motif, which binds to Atg8. Based on amino acid similarity, Atg32 has no mammalian homologue.

In mammals, mitophagy is involved in mitochondria elimination from reticulocytes, which is mediated by NIP3-like protein X (NIX, also known as BNIP3L)^8^. It is also reported that FUNDC1, localized in OMM, is a receptor for hypoxia-induced mitophagy^9^. The OMM kinase, phosphatase and tensin homolog (PTEN)-induced putative kinase protein 1 (PINK1) and the cytosolic E3 ubiquitin ligase Parkin, the mutations of which are causative for hereditary Parkinson’s disease, are known to mediate mitophagy to eliminate damaged mitochondria in many types of cells^10^. Parkin is expressed in most of adult tissues, but some fetal tissues and cell lines including HeLa cells show little or no endogenous Parkin expression^11^,^12^,^13^. Parkin-deficient mice show only mild phenotypes^14^. Thus, it is reasonable to assume that there may be an unknown receptor for mitophagy in mammalian cells. Here, we show that Bcl2-L-13 induces mitochondrial fragmentation and mitophagy in mammalian cells and can function as a mitophagy receptor when it is expressed in yeast.

## Results

### Identification of Bcl2-L-13

In this study, we hypothesized that a mammalian mitophagy receptor will share the following molecular features with Atg32: mitochondrial localization; WXXL/I motifs; acidic amino acid clusters; and single membrane-spanning topology. Using this molecular profile of Atg32 as a search tool, we screened UniProt database (http://www.uniprot.org/) for novel Atg32 functional homologues and identified Bcl-2-like protein 13 (Bcl2-L-13).

**Figure 1:**
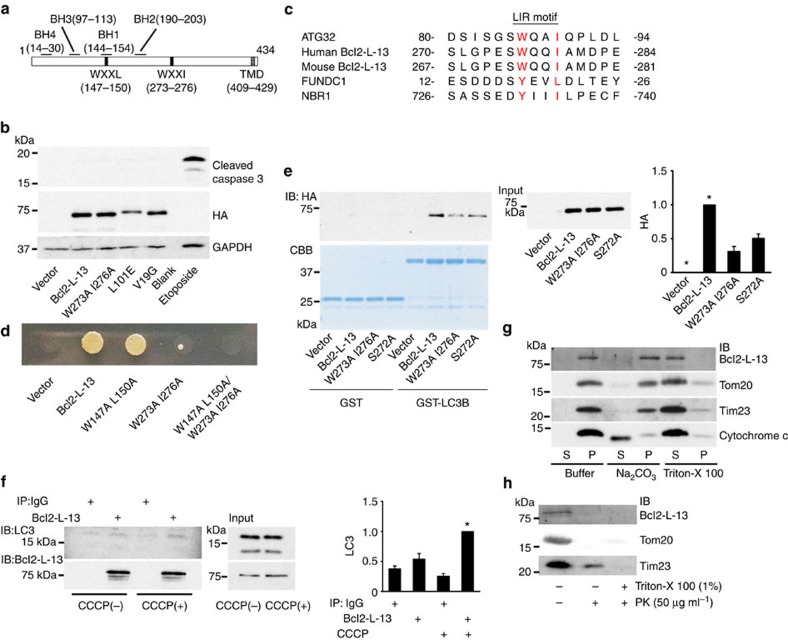
Molecular characteristics of Bcl2-L-13. (**a**) Schematic representation of mouse Bcl2-L-13 domain structure. The predicted BH1-4 domains, WXXL/I motifs and transmembrane domain (TMD) are indicated. (**b**) Bcl2-L-13 failed to induce apoptosis in HEK293A cells. Western blot analysis of cleaved caspase 3 in whole-cell lysates from cells expressing the indicated HA-tagged Bcl2-L-13 constructs. Cell lysates with 100 μM etoposide treatment for 24 h were shown as a positive control. (**c**) Sequence alignment of the WXXL/I motif in Atg32, human and mouse Bcl2-L-13, and human FUNDC1 and NBR1. (**d**) Yeast two-hybrid assay to examine the interaction between LC3B and wild-type or the indicated mutant HA-Bcl2-L-13. (**e**) HEK293A cells were transfected with wild-type or mutant HA-Bcl2-L-13. Forty-eight h after transfection, cells were lysed and incubated with GST-LC3B. The co-precipitated proteins with GST-LC3B were detected by immunoblotting for HA. Coomassie brilliant blue staining was performed to visualize GST or GST-LC3B. Right panel represents densitometric analysis of the band for HA. The value for cells transfected with Bcl2-L-13 in each experiment was set equal to 1 (*n*=3). Results are shown as the mean±s.e.m. *P<0.05 versus all other groups. (**f**) HEK293A cells were treated with 5 μM CCCP and 20 nM bafilomycin A1 for 1 h. Then, cells were lysed and immunoprecipitated with anti-Bcl2-L-13 antibody. Co-precipitated LC3 were detected by immunoblotting. Right panel represents densitometric analysis of the band for LC3. The value for cells treated with CCCP and immunoprecipitated with anti-Bcl2-L-13 antibody was set equal to 1 (*n*=3). Results are shown as the mean±s.e.m. *P<0.05 versus all other groups. (**g**) Mitochondrial fraction from HEK293A cells in the mitochondrial buffer (Buffer) alone or in buffer containing Na_2_CO_3_ or Triton X-100. The pellets (P) and supernatant fractions (S) were immunoblotted with the indicated antibodies. (**h**) Mitochondrial fraction was digested with proteinase K in the absence or presence of Triton X-100 and immunoblotted with the indicated antibodies. A one-way ANOVA followed by Tukey–Kramer’s *post hoc* test was used for statistical analysis.

Mouse Bcl2-L-13 gene (*Bcl2l13*) encodes a protein of 434 amino acids, which contains a C-terminal single transmembrane domain^15^ (Fig. 1a). Bcl2-L-13 is expressed in all tissues and cell lines tested including HeLa cells and localized in mitochondria. It has been known that Bcl2-L-13 contains four conserved Bcl-2 homology domains (BH1-4) and triggers cell death independently of the BH domains. In contrast to the previous report, overexpression of Bcl2-L-13 did not induce the activation of caspase 3 in HEK293 cells (Fig. 1b). Sequence alignment of mouse Bcl2-L-13 revealed 2 WXXL/I motifs at positions 147–150 and 273–276 (Fig. 1a,c).

### Bcl2-L-13 binds to LC3

To examine the interaction of Bcl2-L-13 with LC3, a mammalian homologue of Atg8, a yeast two-hybrid assay was performed between Gal4-fused LC3B and activation domain-fused Bcl2-L-13. The cells expressing LC3B and wild-type Bcl2-L-13 could grow on selective plates (Fig. 1d). We generated Bcl2-L-13 mutants containing amino acid substitution in the WXXL/I motifs and found that Bcl2-L-13 W273A I276A or W147A L150A/W273A I276A, but not W147A L150A had a reduced interaction with LC3B. Consistent with the yeast two-hybrid results, purified glutathione S-transferase (GST)-LC3B could pull down wild-type Bcl2-L-13 overexpressed in HEK293 cells, but Bcl2-L-13 W273A I276A showed decreased binding with LC3 (Fig. 1e), suggesting the second WXXL/I motif at residues 273–276 is a functional LC3-interacting region (LIR)^16^ (Fig. 1c). The mitochondrial uncoupler carbonyl cyanide m-chlorophenylhydrazone (CCCP) has been used to induce mitochondrial damage and mitophagy^17^. CCCP treatment increased the interaction of endogenous Bcl2-L-13 with LC3 in HEK293 cells (Fig. 1f).

### Bcl2-L-13 is an outer mitochondrial membrane protein

To confirm that Bcl2-L-13 is an integral mitochondrial membrane protein, the mitochondrial fraction from HEK293 cells was treated with Na_2_CO_3_, which results in release of soluble and peripheral proteins^18^,^19^ (Fig. 1g). Bcl2-L-13, the outer-membrane protein, Tom20 and the inner-membrane protein, Tim23 were retained in the pellet, whereas cytochrome c was released to the supernatant, suggesting Bcl2-L-13 is an integral mitochondrial membrane protein. Treatment of mitochondrial fraction with proteinase K resulted in almost complete digestion of Bcl2-L-13 and Tom20, but a significant amount of Tim23 remained (Fig. 1h). These results indicate that Bcl2-L-13 is an integral OMM protein. Since the anti-Bcl2-L-13 antibody used in this study recognizes amino acids near the centre of Bcl2-L-13, Bcl2-L-13 has its N-terminus exposed to the cytosol and C-terminus in the intermembrane space.

### Bcl2-L-13 induces mitochondrial fragmentation

**Figure 2:**
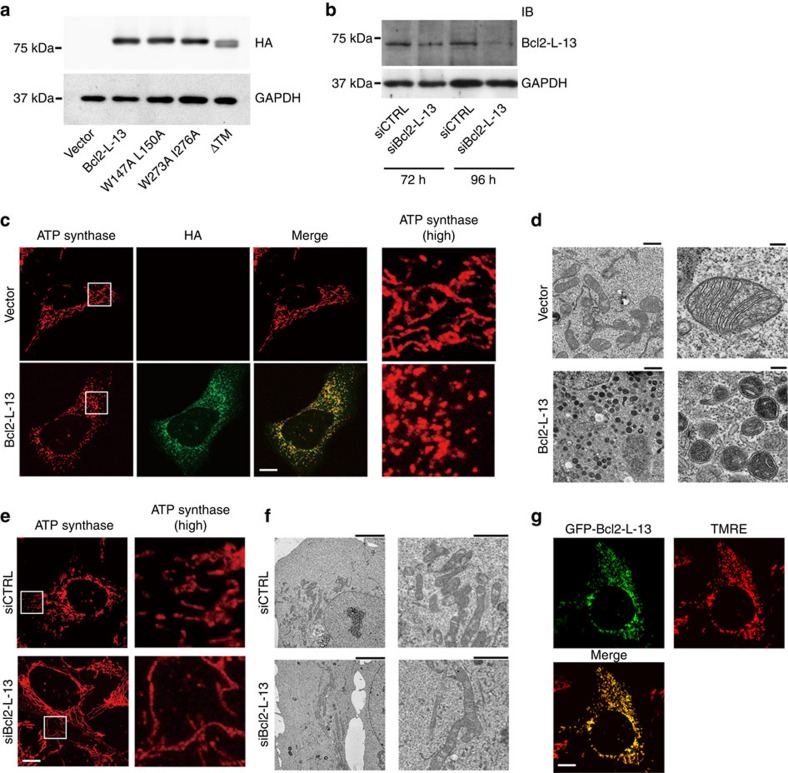
The function of Bcl2-L-13 in mitochondrial dynamics. (**a**,**b**) Expression level of Bcl2-L-13 mutants. Immunoblot of whole-cell lysates from HEK293A cells expressing various HA-tagged Bcl2-L-13 constructs 48 h after transfection (**a**). HEK293A cells were transfected with Bcl2-L-13-specific siRNA (siBcl2-L-13) or with a non-targeting control siRNA (siCTRL), and analysed by immunoblotting with anti-Bcl2-L-13 antibody (**b**). (**c**) Mitochondrial morphology in HEK293A cells transfected with empty vector or HA-Bcl2-L-13 was analysed using anti-ATP synthase and anti-HA antibodies. Boxed areas for ATP synthase staining are shown at higher magnification (high) in the right panels. Scale bar, 10 μm. (**d**) Electron micrographs of mitochondria in HEK293A cells transfected with empty vector or HA-Bcl2-L-13. Images at higher magnification are shown in the right panels. Scale bar, 1 μm in the left panels and 200 nm in the right panels. (**e**) Representative z-stack confocal images of mitochondrial morphologies in HEK293A cells transfected with siCTRL or siBcl2-L-13 using anti-ATP synthase antibody. Scale bar, 10 μm. (**f**) Electron micrographs of mitochondria in HEK293A cells transfected with siCTRL or siBcl2-L-13. Images at higher magnification are shown in the right panels. Scale bar, 5 μm in the left panels and 2 μm in the right panels. (**g**) HEK293A cells transfected with GFP-Bcl2-L-13 were strained with TMRE. Scale bar, 10 μm.

To identify functions of Bcl2-L-13, HA-Bcl2-L-13 was exogenously introduced into HEK293 cells or knockdown of endogenous Bcl2-L-13 was performed using small interfering RNA (siRNA) (Fig. 2a,b). HA-Bcl2-L-13 overexpression induced mitochondrial fragmentation (Fig. 2c,d). Bcl2-L-13-induced fragmented mitochondria were seen as numerous small sphere-like structures. Intra-mitochondrial density increased, but their cristae structure appeared to be maintained. The Bcl2-L-13 siRNA decreased the endogenous protein level (Fig. 2b), which induced mitochondrial elongation (Fig. 2e,f), indicating that endogenous Bcl2-L-13 is required for mitochondrial fission. The mitochondrial potential estimated by tetramethylrhodamine ethyl ester (TMRE) was maintained in the fragmented mitochondria (Fig. 2g).

**Figure 3:**
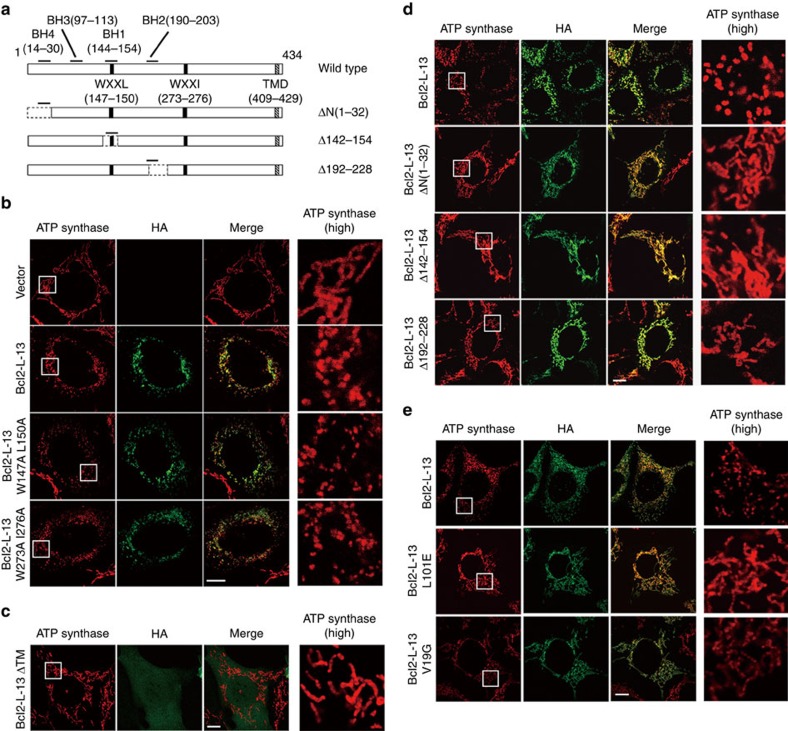
The role of BH domains and LIR motifs in Bcl2-L-13-mediated mitochondrial fragmentation. (**a**) Schematic diagrams of Bcl2-L-13 mutants in the BH domains and LIR. (**b–e**) HEK293A cells transfected with wild-type or mutant HA-Bcl2-L-13 constructs were immunostained for ATP synthase and HA. Boxed areas for ATP synthase staining are shown at higher magnification in the right panels. (**b**) LIR mutants, (**c**) TMD deletion mutant (ΔTM), (**d**) BH domain deletion mutants and (**e**) BH domain amino acid mutants. Scale bar, 10 μm.

We examined the roles of the WXXL/I motifs, transmembrane region and BH domains in Bcl2-L-13-induced mitochondrial fragmentation. Overexpression of WXXL/I mutants containing W147A L150A or W273A I276A substitution induced mitochondrial fragmentation (Figs 2a and 3a,b). The Bcl2-L-13 lacking the transmembrane domain showed diffuse cytoplasmic expression pattern without mitochondrial fragmentation (Fig. 3c). Thus, mitochondrial localization but not LC3 binding is required for Bcl2-L-13-induced fragmentation. A deletion mutant for BH4 (ΔN(1–32)), BH1 (Δ142–154) or BH2 (Δ192–228) domain or an amino acid mutant in BH3 (L101E) or BH4 (V19G) did not induce mitochondrial fragmentation (Fig. 3a,d,e). The point mutations (L101E and V19G) had no effect on apoptosis (Fig. 1b). Thus, all BH1-4 domains are important for Bcl2-L-13-induced fragmentation.

### The relationship between Bcl2-L-13 and Drp1

**Figure 4:**
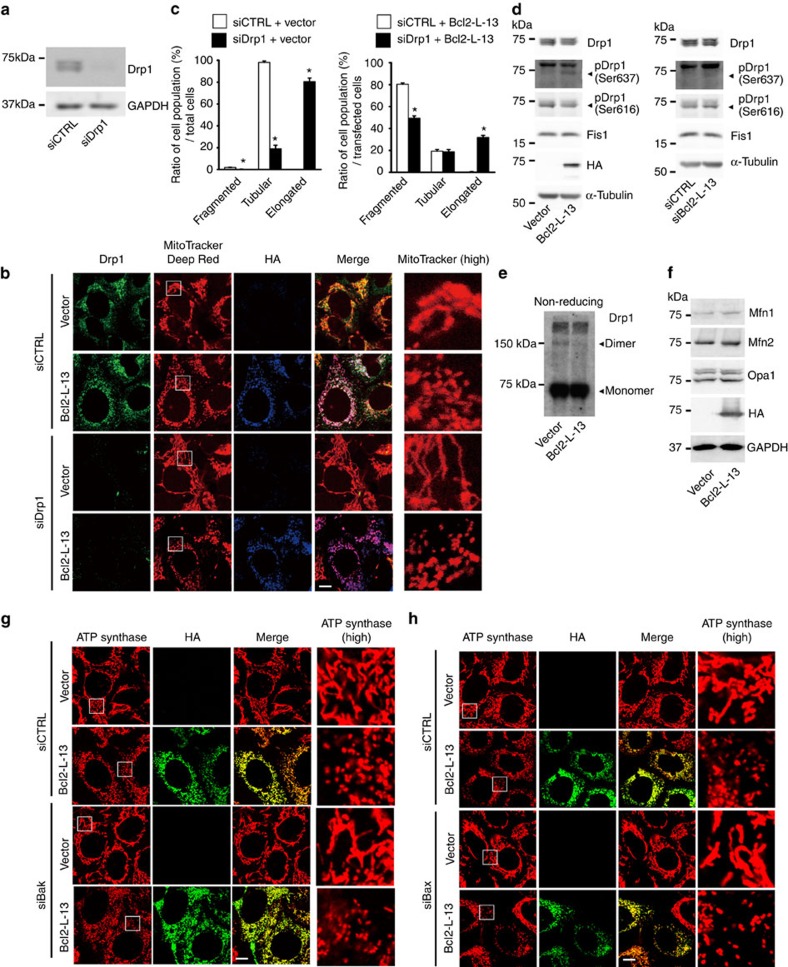
Drp1 is not essential for Bcl2-L-13-induced mitochondrial fragmentation. (**a**) HEK293A cells were transfected with Drp1-specific siRNA (siDrp1) or with a non-targeting control siRNA (siCTRL), and after 48 h analysed by immunoblotting for Drp1. (**b**) Drp1 knockdown HEK293A cells were transfected with empty vector or HA-Bcl2-L-13 for 48 h and then immunostained with the indicated antibodies along with MitoTracker Deep Red. Scale bar, 10 μm. Boxed areas in the MitoTracker Deep Red images are shown at higher magnification (high) in right panels. (**c**) Transfected cells in (**b**) were scored according to their mitochondrial morphology as fragmented, tubular or elongated patterns, and the percentages of distribution are shown in the bar graphs. More than 80 cells were counted for each group (*n*=3). Results are shown as the mean±s.e.m. *P<0.05 versus corresponding control group (Student’s *t*-test). (**d**) Lysates from HEK293A cells transfected with empty vector, HA-Bcl2-L-13 or siBcl2-L-13 were subjected to immunoblotting with the indicated antibodies. α-tubulin was used as a loading control. (**e**) HEK293A cell lysates expressing empty vector or HA-Bcl2-L-13 were subjected to SDS–gel electrophoresis in the absence of 2-mercaptoethanol followed by immunoblotting with anti-Drp1 antibody. (**f**) HEK293A cell lysates expressing empty vector or HA-Bcl2-L-13 were subjected to immunoblotting with the indicated antibodies targeted to proteins involved in mitochondrial fusion. (**g**) Bak knockdown HEK293A cells were transfected with empty vector or HA-Bcl2-L-13 for 48 h and then immunostained with the indicated antibodies. Scale bar, 10 μm. Boxed areas in the ATP synthase staining are shown at higher magnification (high) in the right panels. (**h**) Bax knockdown HEK293A cells were transfected with empty vector or HA-Bcl2-L-13 for 48 h and then immunostained with the indicated antibodies. Scale bar, 10 μm. Boxed areas in the ATP synthase staining are shown at higher magnification (high) in the right panels.

Dynamin-related protein 1 (Drp1) is known to be the master regulator of mitochondrial fission^1^. Mitochondrial fission 1 (Fis1) is a partner protein of Drp1. On the other hand, mitofusins, Mfn1 and Mfn2, together with optic atrophy protein 1 (Opa1) are the core components of the mitochondrial fusion machinery. Knockdown of Drp1 resulted in elongation of mitochondria and overexpression of HA-Bcl2-L-13 was still able to induce mitochondrial fragmentation in Drp1 knockdown cells (Fig. 4a–c). However, the ratio of cells with fragmented mitochondria was reduced in Drp1 knockdown cells. Phosphorylation of Drp1 on Ser637 inhibits mitochondrial fission, while phosphorylation of that on Ser616 promotes mitochondrial fission^20^. Overexpression of Bcl2-L-13 increased the amount of phosphorylated Drp1 on Ser637 and decreased that on Ser616 (Fig. 4d). On the other hand, knockdown of Bcl2-L-13 increased the amount of phosphorylation on Ser616, but not on Ser637, when compared with control siRNA cells. Dimer formation of the dynamin-related proteins such as Drp1 increases their GTPase activity^20^. Overexpression of Bcl2-L-13 led to a decrease in dimer formation of Drp1 (Fig. 4e). These data suggest that Drp1 appeared to compensate Bcl2-L-13-induced changes in mitochondrial morphology. Thus, Drp1 is not essential for Bcl2-L-13-induced mitochondrial fragmentation. Changes in Bcl2-L-13 expression had no effect on the protein level of Fis1 or the molecules involved in fusion such as Mfn1, Mfn2 and Opa1 (Fig. 4d,f). In addition, HA-Bcl2-L-13 was able to induce mitochondrial fragmentation in Bak or Bax knockdown cells (Fig. 4g,h).

### Bcl2-L-13 induces mitophagy

**Figure 5:**
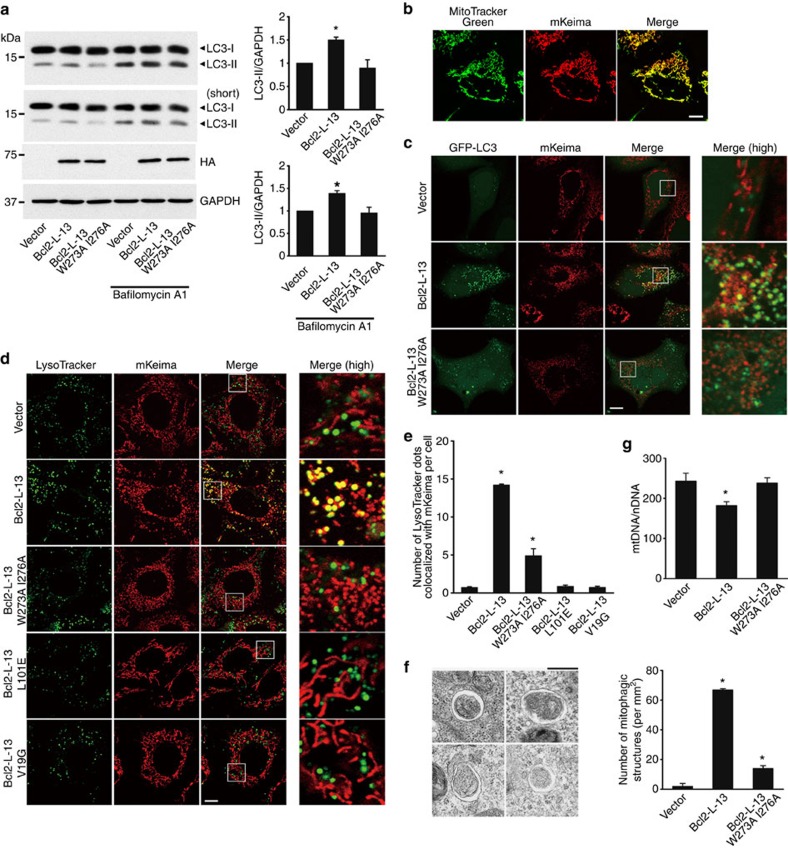
The role of LIR in Bcl2-L-13-induced mitophagy. (**a**) Immunoblots for LC3. HEK293A cells transfected with the indicated vectors were treated with or without 20 nM of bafilomycin A1 for 1 h. The two blots from the top were originated from the same transfer membrane, but exposure time for the second blot (short) was shorter than that for the top blot. Right upper and lower panels represent densitometric analysis of the ratio of LC3-II to GAPDH in non-treated groups on the top blot and that in bafilomycin A1-treated groups on the second blot, respectively. The value for cells transfected with empty vector in each experiment was set equal to 1 (*n*=3). (**b**) mKeima expressing HEK293A cells were excited using 559 nm laser. Scale bar, 10 μm. (**c**) Colocalization of autophagosomes and mitochondria in mKeima expressing cells transfected with GFP-LC3 and wild-type or LIR mutant HA-Bcl2-L-13. Forty-eight h after transfection, cells were treated with E64d and pepstatin A for 4 h prior to analysis. Images in the box at higher magnification are shown in the right panels. Scale bar, 10 μm. (**d**) Colocalization of lysosomes and mitochondria in mKeima-expressing HEK293A cells transfected with the indicated vectors for 48 h. Cells were stained with LysoTracker before microscopic analysis. Scale bar, 10 μm. Images in the box at higher magnification are shown in the right panels. (**e**) Quantification of the number of LysoTracker-positive dots colocalized with mKeima in cells from (**d**). More than 60 cells were counted for each group (*n*=3). (**f**) Electron micrographs of autophagosomes engulfing mitochondria in an HEK293A cell transfected with HA-Bcl2-L-13. E64d and pepstatin A were added 4 h before fixation. Scale bar, 500 nm. Right graph shows the number of mitophagic structures, which were counted in cell areas of more than 0.165 mm^2^ for each group (*n*=3). (**g**) Mitochondrial DNA (mtDNA) normalized to nuclear DNA (nDNA) (*n*=7). All quantitative results are shown as the mean±s.e.m. *P<0.05 versus all other groups. A one-way ANOVA followed by Tukey–Kramer’s *post hoc* test was used for statistical analysis.

Then, we investigated the role of Bcl2-L-13 in mitophagy. Conversion of LC3-I to LC3-II is an essential step for autophagosome formation^21^. HA-Bcl2-L-13 increased LC3-II protein level (left top blot and right upper panel in Fig. 5a). To assess the autophagy flux, we treated cells with a lysosomal inhibitor, bafilomycin A1. Treatment with bafilomycin A1 led to an increase in protein level of LC3-II. For quantitative analysis, we exposed the transfer membrane to the film for a shorter period (the second blot from the top blot in Fig. 5a). The level of LC3-II in HA-Bcl2-L-13 overexpressing cells was higher than that in cells transfected with empty vector or Bcl2-L-13 W273A I276A after treatment with bafilomycin A1 (right lower panel in Fig. 5a). This indicates that the increase in LC3-II level in HA-Bcl2-L-13 overexpressing cells is not because of reduced autophagosome turnover, but increased autophagic flux. We produced a stable cell line of HEK293 expressing mitochondrial targeted Keima (mKeima), a coral-derived acid-stable lysosomal proteases-resistant fluorescent protein^22^. We used the excitation of mKeima at 559 nm that causes emission when the molecule is in acidic compartments such as lysosome as well as in neutral compartments^22^ (Fig. 5b). We transfected the cells with HA-Bcl2-L-13 and GFP-LC3 and incubated with protease inhibitors to derive sufficient number of autophagosomes or autolysosomes for analysis. Bcl2-L-13 increased the number of LC3- and mKeima-positive dots (Fig. 5c). Next, mKeima-expressing HEK293 cells were transfected with HA-Bcl2-L-13 and stained with LysoTracker Green before microscopic analysis (Fig. 5d,e). HA-Bcl2-L-13 increased the number of LysoTracker- and mKeima-positive dots. These results indicate that mitochondria were engulfed in autophagosomes or autolysosomes. Ultrastructural analysis of HA-Bcl2-L-13 expressing HEK293 cells revealed double-membrane vacuoles containing a single mitochondrion-like structure, but little cytoplasm (Fig. 5f). HA-Bcl2-L-13 significantly reduced mitochondrial DNA amount (Fig. 5g). These indicate that Bcl2-L-13 induced mitophagy. The mutations in the LIR domain reduced mitophagic activity (Fig. 5a,c–g). The mutants in the BH domains were unable to induce mitochondrial fragmentation and subsequent mitophagy (Fig. 5d,e). Thus, Bcl2-L-13 induces mitophagy through the interaction with LC3.

### Bcl2-L-13 induces mitophagy in Parkin-independent manner

**Figure 6:**
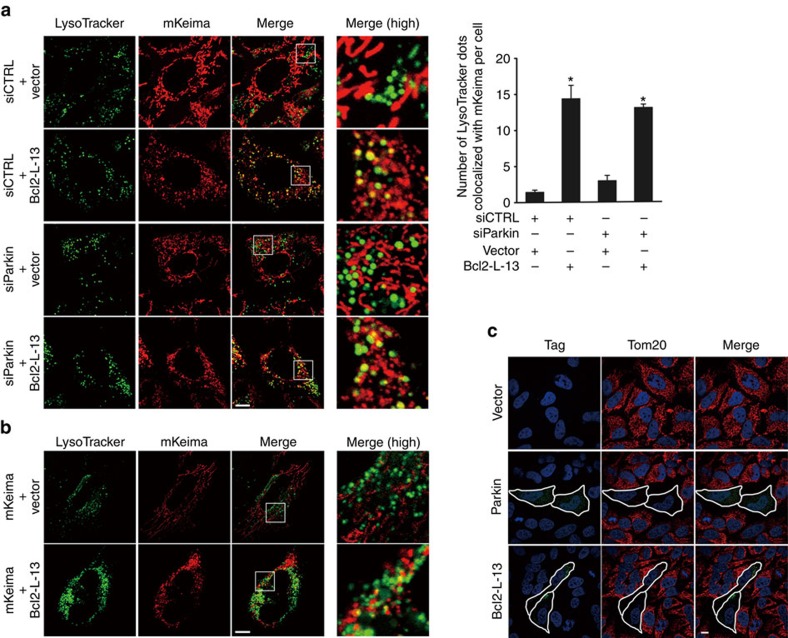
Bcl2-L-13-induced mitophagy is Parkin-independent. (**a**) Parkin knockdown in HEK293A cells stably expressing mKeima was achieved by transfection with control siRNA (siCTRL) or Parkin-specific siRNA (siParkin). Then, the cells were transfected with empty vector or HA-Bcl2-L-13 and stained with LysoTracker. Scale bar, 10 μm. Images at higher magnification of areas in the box are shown in right panels. Right graph, quantification of the number of LysoTracker dots colocalized with mKeima. More than 30 cells were counted for each group (*n*=3). Values represent the mean±s.e.m. *P<0.05 versus siCTRL- or siParkin- and vector-transfected cells (A one-way ANOVA and Tukey–Kramer’s *post hoc* test). (**b**) HeLa cells transfected with pMT-mKeima-Red and the indicated plasmids for 48 h were stained with LysoTracker. Scale bar, 10 μm. (**c**) Selective mitochondria elimination by Parkin or Bcl2-L-13. HeLa cells expressing FLAG-Parkin or HA-Bcl2-L-13 were incubated with 10 μM CCCP for 48 h. Cells were stained with DAPI and antibodies for Tom20 and tag such as FLAG (Parkin) or HA (Bcl2-L-13). Outlines demarcate the edges of transfected cells. Scale bar, 10 μm.

To examine whether Bcl2-L-13-induced mitophagy is coupled to the PINK1/Parkin-mediated pathway, we expressed HA-Bcl2-L-13 in Parkin knockdown HEK293 cells harbouring mKeima. Bcl2-L-13 induced a similar level of mitophagy in Parkin knockdown HEK293 and control cells, as indicated by the number of LysoTracker- and mKeima-positive dots (Fig. 6a). Furthermore, Bcl2-L-13 was able to induce mitophagy in HeLa cells, which reportedly lack a functional *Parkin* gene^12^ (Fig. 6b). It has been reported that the mitochondria were maintained after adding CCCP in HeLa cells, whereas few mitochondria remained detectable in Parkin expressing HeLa cells, assessed by immunocytochemistry using anti-Tom20 antibody^17^. We confirmed the effect of Parkin on CCCP-treated HeLa cells (Fig. 6c). Similar selective mitochondrial elimination by CCCP treatment was observed in Bcl2-L-13 expressing HeLa cells. These indicate that Parkin is not necessary for Bcl2-L-13 to induce mitophagy.

### Bcl2-L-13 functions as a mitophagy receptor in yeast

**Figure 7:**
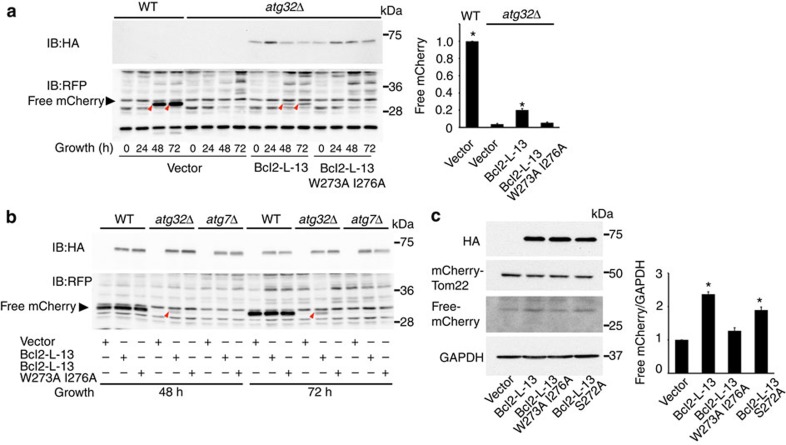
Bcl2-L-13 can substitute for Atg32 to induce mitophagy. (**a,b**) Yeast transfected with wild-type or LIR mutant HA-Bcl2-L-13 or empty vector were collected at the indicated time points after induction of mitophagy and subjected to western blotting for mCherry. The red arrows depict free mCherry generated by mitophagy. Yeast strains are wild-type (WT) or *atg32*Δ in (**a**) or *atg32*Δ or *atg7*Δ derivatives in (**b**) expressing a mitochondrial matrix-targeted DHFR-mCherry. Quantitative analysis for free mCherry in (**a**) is shown in the right graph (*n*=3). The value for wild-type yeast strain was set equal to 1. Results are shown as the mean±s.e.m. *P<0.05 versus all other groups. (**c**) HEK293 cells were transfected with mCherry-Tom22 and wild-type or the indicated mutant HA-Bcl2-L-13. Seventy-two h after transfection, cells were lysed and subjected to western blotting for mCherry. Processed mCherry-Tom22 was detected as free mCherry. Quantitative analysis for free mCherry is shown in the right graph (*n*=3). The value for vector transfected cells was set equal to 1. Results are shown as the mean±s.e.m. *P<0.05 versus all other groups. A one-way ANOVA followed by Tukey–Kramer’s *post hoc* test was used for statistical analysis.

Atg32 is essential for mitophagy under respiratory conditions^6^,^7^. Yeast cells expressing the mitochondrial matrix-targeted dehydrofolate reductase-mCherry protein (mito-dihydrofolate reductase (DHFR)-mCherry) were grown under respiratory conditions^23^. It has been reported that mitophagy in yeast is strongly activated in cells at stationary phase, which seems to be triggered by oxidative stress^6^. Upon mitophagy, this fusion protein is transported and processed to generate free mCherry in the vacuole. In yeast lacking Atg32, the processing of mito-DHFR-mCherry barely occurred. We replaced the transmembrane domain in Bcl2-L-13 with mitochondrial tail-anchor (TAmito) domain derived from an authentic outer membrane protein to facilitate mitochondrial localization of the expressed protein in yeast^23^. Surprisingly, expression of Bcl2-L-13 (1–407)-TAmito in *atg32*Δ yeast generated free mCherry, suggesting partial restoration of the ability to induce mitophagy, but the LIR mutant did not (Fig. 7a). Furthermore, Bcl2-L-13 failed to induce mitophagy in *atg7*Δ (Fig. 7b), suggesting Bcl2-L-13-induced mitophagy is mediated through known autophagy molecular machinery. These results indicate that Bcl2-L-13 can substitute for Atg32 in yeast. To confirm that Bcl2-L-13 induces mitophagy in mammalian cells, we generated a fusion construct of mCherry and Tom22 to target mCherry to mitochondria, and transfected HEK293 cells with mCherry-Tom22 and wild-type or the indicated mutant HA-Bcl2-L-13. Overexpression of Bcl2-L-13 generated free mCherry in HEK293 cells (Fig. 7c). The protein level of processed mCherry in cells transfected with Bcl2-L-13 was more than that in Bcl2-L-13 W273A I276A overexpressing cells.

### The physiological significance of Bcl2-L-13

**Figure 8:**
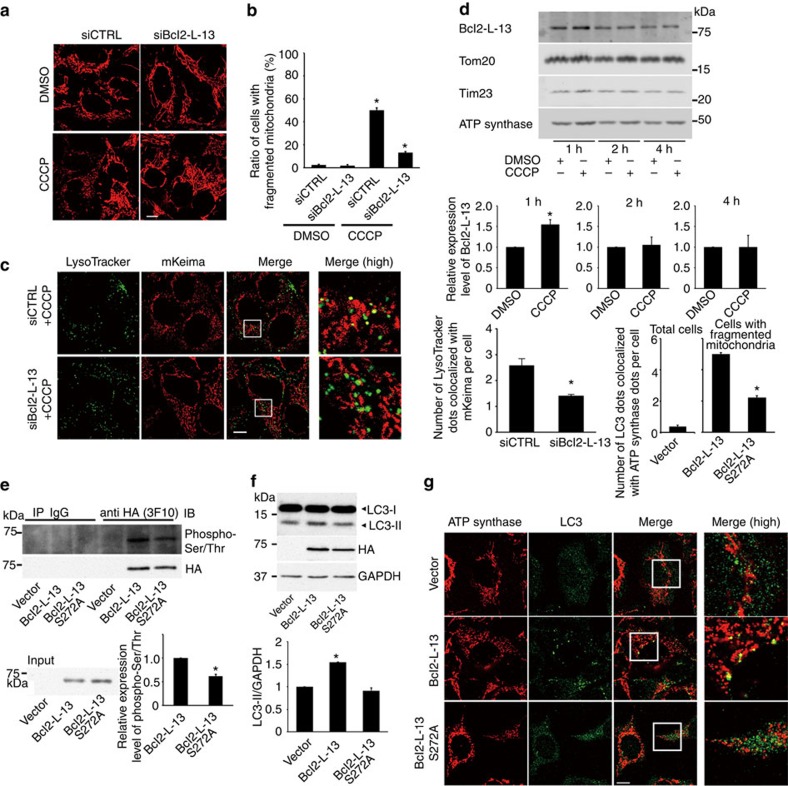
Physiological role of Bcl2-L-13 in mitochondrial homeostasis. (**a**,**b**) HEK293A cells transfected with siRNA were incubated with 5 μM CCCP for 4 h and stained with anti-ATP synthase antibody. Mitochondrial morphology was estimated in (**b**). More than 70 cells were counted for each group (*n*=3). *P<0.05 versus all other groups. Scale bar, 10 μm. (**c**) HEK293A cells expressing mKeima were transfected with siBcl2-L-13, treated with 5 μM CCCP for 4 h and stained with LysoTracker. Quantitative analysis for LysoTracker-positive dots colocalized with mKeima is shown in the right graph. More than 50 cells were counted for each group (*n*=3). *P<0.05 versus control. (**d**) HEK293A cells were treated with 5 μM CCCP for the indicated time and mitochondrial fraction was subjected to immunoblotting (*n*=3). Data were normalized to the corresponding protein level of Tom20 and the value treated with DMSO at the indicated time was set equal to 1. *P<0.05 versus control. (**e**) Forty-eight h after transfection, cells lysates were immunoprecipitated with HA antibody. The phosphorylation was detected using anti-Phospho-Serine/Threonine antibody. Lower panel represents densitometric analysis of phospho-Ser/Thr (*n*=3). *P<0.05 versus Bcl2-L-13. (**f**) Immunoblot for LC3. Lower panel represents densitometric analysis of LC3-II-to-GAPDH ratio (*n*=3). *P<0.05 versus all other groups. (**g**,**h**) Colocalization of autophagosomes and mitochondria in HEK293A cells. After 48 h of transfection, cells were treated with E64d and pepstatin A for 4 h and stained with anti-LC3 and anti-ATP synthase antibody. Images in the box at higher magnification are shown in the right panels. The number of LC3 dots colocalized with ATP synthase per cell showing mitochondrial fragmentation in wild-type or mutant Bcl2-L-13 overexpressing cells in (**h**). At least 30 cells were counted for each group (*n*=3). Scale bar, 10 μm. *P<0.05 versus Bcl2-L-13. Quantitative results are shown as the mean±s.e.m. Student’s *t*-test (**c**,**d**,**e**,**h**) or a one-way ANOVA followed by Tukey–Kramer’s *post hoc* test (**b**,**f**) was used for statistical analysis.

We next investigated the physiological significance of endogenous Bcl2-L-13 in mitochondrial dynamics and mitophagy. CCCP induced fragmentation of mitochondria in HEK293 cells (Fig. 8a,b) and mitophagy in mKeima-expressing HEK293 cells (Fig. 8c). Knockdown of Bcl2-L-13 attenuated CCCP-induced fragmentation and mitophagy, indicating that endogenous Bcl2-L-13 plays an important role in CCCP-induced mitochondrial fragmentation and mitophagy.

**Figure 9:**
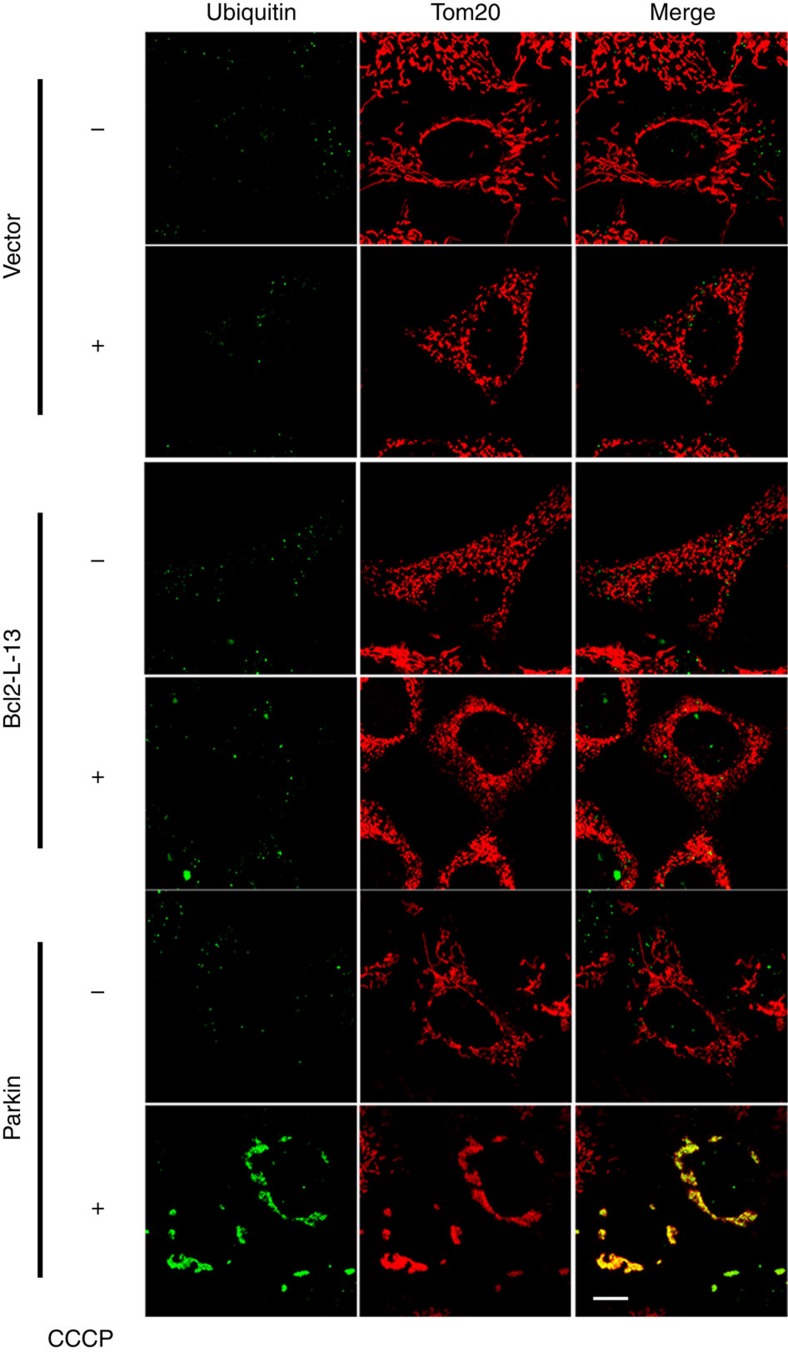
Ubiquitination is not involved in Bcl2-L-13-induced mitophagy. HEK293A cells expressing FLAG-Parkin or HA-Bcl2-L-13 were incubated with 10 μM CCCP for 3 h. Cells were stained antibodies for Tom20 and ubiquitin. Scale bar, 10 μm.

Then, we attempted to elucidate the activation mechanism of Bcl2-L-13 to induce mitochondrial fragmentation and mitophagy. Atg32 is temporally upregulated to induce mitophagy and subsequently degraded^6^,^7^. The Atg11-Atg32 interaction is believed to be the initial molecular step, in which the autophagic machinery recognizes mitochondria as a cargo. Phosphorylation of the Ser-114, close to Atg8-interaction site (amino acid residue 86–89), in Atg32 mediates the Atg11–Atg32 interaction and mitophagy^24^,^25^. Thus, induction of the protein and/or post-translational modification of Bcl2-L-13 might regulate its function. The protein level of Bcl2-L-13 was increased 1 h after CCCP treatment compared with control, then returned to baseline thereafter (Fig. 8d). Overexpression of Bcl2-L-13 induced its Ser/Thr phosphorylation (Fig. 8e). We mutated Ser272 to Ala in Bcl2-L-13, which is close to the second LIR motif. The phosphorylation level of Bcl2-L-13 S272A was significantly attenuated compared with control. Bcl2-L-13 S272A showed less ability for binding with LC3 (Fig. 1e). Overexpression of Bcl2-L-13 S272A induced mitochondrial fragmentation, but decreased the LC3-II protein level and the number of LC3 dots colocalized with ATP synthase (Fig. 8f–h). The amount of processed mitochondrial targeted mCherry in Bcl2-L-13 S272A overexpressing cells was less than that in cells transfected with wild-type Bcl2-L-13 (Fig. 7c). Another possible mechanism to regulate the function of Bcl2-L-13 will be ubiquitination. CCCP induced ubiquitination of mitochondria in Parkin overexpressing cells as previously reported^26^, but not in Bcl2-L-13 overexpressing cells (Fig. 9), excluding the involvement of ubiquitination in the regulation mechanism underlying the functions.

## Discussion

Although Atg32 is essential for as mitophagy in yeast^6^,^7^, no mammalian homologue has been identified. Here, we demonstrate that Bcl2-L-13 is involved in mitophagy as well as mitochondrial fragmentation. Most surprisingly, Bcl2-L-13 exhibited the ability to compensate the function of Atg32 in yeast.

In contrast to our results, Kataoka *et al*.^15^ reported that overexpression of Bcl2-L-13 resulted in caspace-3 activation and cytochrome c release from mitochondria in HEK293T cells. It has been reported that Bcl2-L-13 shows no interaction with either anti-apoptotic (Bcl-2, Bcl-xL, Bcl-w, A1, MCL-1, E1B-19K and BHRF1) or pro-apoptotic (Bax, Bak, Bik, Bid, Bim and Bad) members of the Bcl-2 family, even though it has BH1-4 domains^15^. Although we do not know the exact reason for the discrepancy, some non-specific effects of its overexpression may result in the induction of apoptosis.

Our results indicate that Bcl2-L-13 is localized to OMM and interacts with LC3 through the conserved LIR sequence. In yeast, Atg32 interacts with Atg8 and Atg11 (refs 6, 7), suggesting Atg32 recruits Atg8 and Atg11 and they and other core Atg proteins cooperatively generate isolation membranes surrounding mitochondria. Interestingly, there is no known mammalian homologue of Atg11 (ref. 27). Although the detailed mechanism how Bcl2-L-13 mediates mitophagy is not entirely understood, we can speculate that Bcl2-L-13 recruits LC3 to the surface of mitochondria, leading to the formation of mitochondria-specific autophagosomes (mitophagosomes). Bcl2-L-13 may bind to an unidentified mammalian homologue of Atg11 or may play a role as a scaffold in a similar fashion with Atg11.

Mitophagy is closely linked to mitochondrial dynamics. Thus, it is possible that Bcl2-L-13-induced mitophagy is a consequence of mitochondrial fragmentation. However, our results showing that the mutation in the LIR motif attenuated mitophagy without affecting mitochondrial fragmentation and that Bcl2-L-13 induced mitophagy in Atg32-deficient yeast strongly indicate that Bcl2-L-13 has the dual effects. The molecular mechanisms of Bcl2-L-13-mediated mitochondrial fragmentation remain to be elucidated. Mitochondria exist largely as an extended, reticular structure. For engulfment of mitochondria by isolation membranes, the mitochondrial size will be an issue. In yeast, Atg11 recruits Dnm1 (a yeast homologue of Drp1), the fission machinery to drive mitophagy, suggesting that Atg 11 plays a role as a scaffold that recruits the fission components in addition to its role in connecting the damaged mitochondria with the autophagy machinery and mitochondrial fragmentation and mitophagy occur in a coordinated manner^4^. Thus, the mitochondria destined for degradation will be selected first, and then the fission machinery would be recruited to drive the separation of these mitochondrial from the mitochondrial network and finally the fragmented mitochondria are degraded mediated through autophagy^4^. Since Bcl2-L-13 has the dual effects, the coordination will operate more efficiently. We showed that BH1-4 motifs are involved in Bcl2-L-13-induced fragmentation. Identification of a binding protein to the BH domains in Bcl2-L-13 may elucidate a molecular mechanism underlying the coordinated interaction between fission and mitophagy.

CCCP is the most popular stimulus to induce mitochondrial damage in cell biological research, which induces mitochondrial fragmentation and mitophagy^17^, although the ability of CCCP to induce mitophagy is not so potent as it decreases the total amount of mitochondrial protein level in cells^28^ (Fig. 8d). We showed that Bcl2-L-13 is essential for CCCP-induced mitochondrial fragmentation and mitophagy, suggesting the physiological importance of Bcl2-L-13. This leads to the question of how Bcl2-L-13 is related to known pathways for mitochondrial fragmentation and mitophagy. Our results indicate that Drp1 is not essential for Bcl2-L-13-induced mitochondrial fragmentation. However, we found the existence of elongated mitochondria and reduced ratio of cells with fragmented mitochondria in Drp1 knockdown cells expressing Bcl2-L-13. These results suggest that there may be some interaction between the two pathways. In addition, we showed that Parkin is not necessary for Bcl2-L-13 to induce mitophagy. The Parkin-deficient mice have only modest phenotypes^14^,^29^ and Parkin is not expressed in all cell types, suggesting the presence of alternate pathways of mitophagy. NIX and FUNDC1 mediate mitophagy in mammalian cells^8^,^9^,^30^. These are involved in specific types of mitophagy; the former for mitochondrial elimination from reticulocytes, the latter for hypoxia-induced mitophagy. However, we are not able to exclude the possibility that Bcl2-L-13 can cooperate with NIX and/or FUNDC1 for mitophagy. Germ-line gene ablation of Bcl2-L-13 will provide its *in vivo* physiological role in mitochondrial quality and quantity control.

The molecular mechanism underlying the activation of Bcl2-L-13 to induce mitochondrial fragmentation and mitophagy remains to be elucidated. The induction of the protein might regulate mitochondrial fragmentation and/or mitophagy. Furthermore, the Ser272 phosphorylation of Bcl2-L-13 regulates mitophagy, but not mitochondrial fragmentation. When mitochondrial fragmentation is induced, unknown kinase(s) activates the Bcl2-L-13 by phosphorylation of its Ser residue close to the LIR domain to allow recruitment of mitophagy machinery.

Dysregulation of mitophagy is implicated in the development of many chronic diseases including neurodegenerative diseases, metabolic diseases and heart failure^3^. Our study will provide a novel insight into molecular mechanisms of the pathogenesis of such diseases.

## Methods

### Antibodies

Anti-Tim23 (1/1,000), anti-Tom20 (for western blots, 1/1,000, for immunofluorescence, 1/200), anti-Bcl2-L-13 (1/1,000) (Proteintech), anti-cytochrome c (1/1,000) (BD biosciences), anti-Bcl2-L-13 (1/500), anti-GAPDH (1/1,000), anti-Fis1 (1/1,000), anti-α-tubulin (1/1,000) (Abcam), anti-LC3B (for western blots, 1/1,000, for immunofluorescence, 1/100), anti-HA (for western blots, 1/1,000, for immunofluorescence, 1/200), anti-Drp1Ser637 (1/500), anti-Drp1Ser616 (1/1,000), anti-Mfn2 (1/1,000), anti-cleaved caspase 3 (1/1,000) (Cell Signaling Technology), anti-ATP synthase subunit beta (for western blots, 1/1,000, for immunofluorescence, 1/200) (Life Technologies), anti-Drp1 (for western blots, 1/1,000, for immunofluorescence, 1/100), anti-Opa1 (1/1,000) (BD Transduction Laboratories), anti-FLAG (for immunofluorescence, 1/200) (Sigma), anti-Mfn1 (1/1,000) (Abnova), anti-ubiquitin (for immunofluorescence, 1/200) (Enzo Life Science), anti-Phospho-Serine/Threonine (Upstate), anti-mCherry (1/500) (Clontech) and anti-RFP^23^ (1/1,000) antibodies were used in this study.

### Cell culture and transfection

HEK293A and HeLa cells were purchased from ATCC. HEK293A cells were grown in Dulbecco’s modified Eagle’s medium (Sigma) supplemented with 10% fetal bovine serum (Sigma) and 1% penicillin-streptomycin (Life Technologies) at 37 °C under 5% CO_2_. Transient transfections were performed using Lipofectamine 2000 (Life Technologies) or ScreenFect A (Wako) according to the manufacturer's instructions. After 48 h of transfections, cells were subjected to analysis, unless otherwise indicated. Mitophagy was induced in HEK293A and HeLa cells by the treatment with 5 and 10 μM of CCCP for the indicated time, respectively. Control cells were treated with the vehicle, dimethylsulphoxide (DMSO).

### siRNA-mediated knockdown

Cells were transfected with siRNA for knockdown of Parkin (30 nM stealth RNAi (Life Technologies)), Drp1 (20 nM BTsiMAX (BONAC)), Bak (2.5 nM Silencer Select (Life Technologies)), Bax (25 nM ON-TARGET plus (Dharmacon)) or Bcl2-L-13 (40 nM BTsiMAX) using ScreenFect A or RNAiMax (Life Technologies). Non-targeting siRNA control was obtained from BONAC. After 72 h of transfection, cells were subjected to analysis, unless otherwise indicated.

### Plasmid constructions

Constructs were obtained by conventional restriction enzyme-based cloning. Site-directed mutagenesis was performed using the QuikChange Site-Directed Mutagenesis Kit according to the supplier’s instructions (Agilent Technologies).

cDNA encoding LC3B or Bcl2-L-13 was obtained by RT-PCR from C57B/6J mouse heart total RNA with LC3B forward (5′-GAATTCATGCCGTCCGAGAAGACCTTC-3′) and reverse (5′-GAATTCGTCCGCTGGTAACATCCCTT-3′) primers, and Bcl2-L-13 forward (5′-AGCAGAATTCATGGCGTCCTCTACGACTGC-3′) and reverse (5′-AGCAGAATTCTTACTTTCTTCTTAAAGCCAGTGCA-3′, Rambo-R) primers, respectively. The amplified fragments were directly inserted into pCR2.1-TOPO (Invitrogen) or pTA2 (Takara) and subcloned into EcoR I site of pGBKT7 or pGADT7. pGADT7-Bcl2-L-13 W147A L150A, W273A I276A, W147A L150A/W273A I276A were created by PCR-based site-directed mutagenesis using pGADT7-Bcl2-L-13 as a template.

To obtain pGEX-LC3B fragment, mouse LC3B was amplified from pGBKT7-LC3B with forward (5′-AGCAGGATCCGCCGCCATCATGGAGGAGCAGAAGCTG-3′) and reverse (5′-CTAGTTATGCGGCCGCTGCAGG-3′) primers. The amplified fragment was directly inserted into pCR2.1-TOPO and then subcloned into EcoR I site of pGEX-6P-2.

pRS316-Atg32(1–388)-TAmito-3HA was previously described^23^. To obtain p416GPD-Bcl2-L-13(1–407)-TAmito, a fragment of Bcl2-L-13(1–407) was amplified from pGADT7-Bcl2-L-13 with forward (5′-AGCAACTAGTGCCGCCATGGAGTACCCATACGACGTA-3′, Rambo-F) and reverse (5′-AGCATCTAGACTTGCCCTCGGCGGGCAGGCCACT-3′) primers. The amplified fragment was directly inserted into pCR2.1-TOPO and then subcloned into pRS316-atg32(1–388)-TAmito, in replacement for atg32(1–388). Finally Bcl2-L-13-TAmito fragment was subcloned into p416GPD. To generate p416GPD-Bcl2-L-13 W273A I276A (1–407)-TAmito, a fragment flanking the LIR was obtained from pcDNA3.1-Bcl2-L-13 W273A I276A by Sma I digestion and subcloned into the corresponding site of p416GPD-Bcl2-L-13(1–407)-TAmito.

To obtain pcDNA3.1-HA-Bcl2-L-13, Bcl2-L-13 including HA tag was amplified from pGADT7-Bcl2-L-13 with forward Rambo-F and reverse (5′-AGCATCTAGACTTGCCCTCGGCGGGCAGGCCACT-3′) primers. The amplified fragment was subcloned into BamH I/Xho I site of pcDNA3.1. To obtain pcDNA3.1-HA-Bcl2-L-13 ΔTM fragment, a stop codon after 408 Ala in Bcl2-L-13 was introduced by PCR from pcDNA3.1-HA-Bcl2-L-13 with forward Rambo-F and reverse (5′-AGCATCAGGCCTTGCCCTCGGCGGG CAGGCCACT-3′) primers. The amplified fragment was directly inserted into pCR2.1-TOPO and then subcloned into BamH I/Xho I site of pcDNA3.1. To create pcDNA3.1-HA-Bcl2-L-13 ΔN(1–32), a fragment of Bcl2-L-13 was obtained from pcDNA3.1-HA-Bcl2-L-13 with forward (5′-AGCAGAATTCATGGGTCCCTCACCCCCAGGAGTT-3′) and reverse Rambo-R primers. The amplified fragment was directly inserted into pCR2.1-TOPO and subcloned into EcoR I site of pcDNA3.1-HA-Bcl2-L-13. To create pcDNA3.1-HA-Bcl2-L-13 Δ142–154 and Δ192–228, N-terminal or C-terminal side of the deletion site was amplified from pcDNA3.1-HA-Bcl2-L-13 with the following primer pairs: Δ142–154 N, forward (Rambo-F) and reverse (5′-AGCACTGCAGTCTCCACTGTACATTC-3′) primers, Δ142–154 C: forward (5′-AGCACTGCAGTTTTGCTACAACACCTG-3′) and reverse Rambo-R primers, Δ192–228 N: forward Rambo-F and reverse (5′-AGCATCCGGAGATGAACTCTGCGGCGTGCTC-3′) primers, Δ192–228 C: forward (5′-AGCATCCGGACAAGTTAGTCCCCCT-3′) and reverse Rambo-R primers. The amplified fragments were directly inserted into pCR2.1-TOPO and subcloned into EcoR I site of pcDNA3.1-HA-Bcl2-L-13. pcDNA3.1-HA-Bcl2-L-13 L101E, V19G and S272A were obtained by site-directed mutagenesis of pcDNA3.1-HA-Bcl2-L-13. Parkin cDNA was created by PCR from C57B/6 J mouse heart cDNA with Parkin forward (5′-GCCCGGTGACCATGATAGTG-3′) and reverse (5′-TTTCCCTTGAGGTTGTGCGT-3′) primers. The amplified fragments were directly inserted into pCR2.1-TOPO and cloned into Not I/Xba I site of pCMV-FLAG4.

To obtain GFP-Bcl2-L-13, a fragment from pcDNA3.1-HA-Bcl2-L-13 was cloned into BspE1 and Sal1 site of pEGFP-C1. To obtain pmCherry-Tom22, cDNA encoding Tom22 was obtained by RT-PCR from C57B/6J mouse heart total RNA with forward (5′-TCCGGAATGGCCGCCGCCGTCGCTGCAGCC-3′) and reverse (5′-CTACATCTTTCCAGGAAGTGGAGG-3′) primers. The amplified fragments were directly inserted into pCR2.1-TOPO and subcloned into BspE1 site of pmCherry-C1 (Clontech).

All plasmid constructs were verified by restriction digestion and/or DNA sequencing. pMT-mKeima-Red was purchased from MBL. pEGFP-LC3 was obtained from Prof. Noboru Mizushima^21^.

### Establishment of mKeima stable expression cell line

The pMT-mKeima-Red plasmid was transfected into HEK293A cells using calcium phosphate method. After 48 h, the cells were passaged and 1 mg ml^−1^ G418 for selection was added 24 h later. After 14 days, the single colonies were transferred to 24-well plates and expanded.

### Yeast two-hybrid assay

Bcl2-L-13 or its mutants containing W147A L150A or W273A I276A substitution and LC3B were cloned into pGADT7 and pGBKT7, respectively. The cloned constructs were co-introduced into AH109 yeast strain using lithium acetate/polyethylene glycol with herring testis carrier DNA. The transformants were spotted on agar plates containing a synthetic dropout medium (Clontech) lacking Leu and Trp for maintenance of the plasmids, and those additionally lacking His and Ade to suppress background, and grown at 30 °C for 4 days.

### Mitophagy assay in yeast and mammalian cells

The cytosolic domain of Bcl2-L-13 was fused in frame with a tail-anchor domain (amino acids 618–662) of Gem1, an authentic mitochondrial outer membrane protein in yeast^31^. p416GPD-Bcl2-L-13(1–407)-TAmito or the LIR mutant construct was introduced into yeast strains expressing mitochondrial matrix-targeted DHFR-mCherry using lithium acetate/polyethylene glycol with herring testis carrier DNA^23^. The transformants were plated on SDCA-U (synthetic medium (0.17% yeast nitrogen base without URA) containing 0.5% casamino acids and 2% dextrose) plate for 48 h and streaked out onto the stock plate. For mitophagy induction, cells pregrown to mid-log phase in SDCA-U medium were incubated at 30 °C for 1–3 days in synthetic medium containing 0.5% casamino acids and 0.1% dextrose plus 3% glycerol (SGlyCA). Generation of free mCherry was detected by western blotting. Yeast strains used are KOY1387 (*his3*Δ*1 leu2*Δ*0 met15*Δ*0 ura3*Δ*0 TEFp-mito-DHFR-mcherry::CgHIS3*), KOY1422 (*his3*Δ*1 leu2*Δ*0 met15*Δ*0 ura3*Δ*0 TEFp-mito-DHFR-mcherry::CgHIS3 atg32::kanMX6*) and KOY1424 (*his3*Δ*1 leu2*Δ*0 met15*Δ*0 ura3*Δ*0 TEFp-mito-DHFR-mcherry::CgHIS3 atg7::kanMX6*).

HEK293A cells were transfected with 250 ng of mCherry-Tom22 and 750 ng of wild-type or the HA-Bcl2-L-13 mutants. Seventy-two h after transfection, cells were lysed and subjected to western blotting for mCherry. Processed mCherry-Tom22 was detected as 30 kDa protein band of free mCherry.

### Live cell and immunofluorescence microscopy

HEK293A cells stably expressing mKeima were seeded at 5.0 × 10^4^ ml^−1^ in a glass-based dish (Iwaki) and transfected 24 h later using ScreenFect A according to the manufacturer’s instructions. The cells were imaged 48 h after transfection, otherwise indicated, by FV1000-D (Olympus) equipped with cell culture incubator. To visualize the colocalization of autophagosomes and mitochondria, cells transfected with pEGFP-LC3 and Bcl2-L-13 constructs were treated with E64d (10 μg ml^−1^) and pepstatin A (10 μg ml^−1^) (Peptide Institute) for 4 h prior to analysis. We excited mKeima at 559 nm and collected the emission from 570 to 670 nm to monitor mitochondria.

For immunostaining, cells were plated on sterile coverslip (22 mm diameter). After treatment, cells were fixed with 4% formaldehyde at 37 °C for 10 min, permeabilized with 0.2% Triton X-100 for 15 min, and then blocked with 2% bovine serum albumin for 1 h at room temperature (RT). For immunostaining of endogenous LC3, cells were fixed and permeabilized with methanol for 10 min at −20 °C. Cells were incubated with primary antibodies overnight at 4 °C followed by staining with secondary antibodies for 1 h at RT. After washing, cells were mounted with Vectashield mounting medium (VECTOR Laboratories) and analysed by LSM 510 (Zeiss) or FV1000-D.

To visualize the mitochondria or lysosomes, cells were stained with 100 nM MitoTracker Deep Red FM (Molecular Probes), 100 nM MitoTracker Green or 50 nM LysoTracker Green (Molecular Probes) for 30 min before confocal microscopic analysis or fixation. To evaluate mitochondrial membrane potential, cells were stained with 100 nM TMRE (Molecular Probes) for 30 min before confocal microscopic analysis.

### Electron microscopy

For electron microscopy, cells were first fixed with 2.5% glutaraldehyde (Polysciences) at 37 °C for 30 min and then washed three times with 2.5% glutaraldehyde in 0.1 M phosphate buffer (pH 7.4). For detecting mitophagy, 10 μg ml^−1^ pepstatin A and 10 μg ml^−1^ E64d were added 4 h before fixation. The samples were visualized using transmission electron microscope (Hitachi, H-7650) at 80 kV.

### SDS–PAGE and western blotting

Cells were rinsed in ice-cold PBS and lysed in lysis buffer (50 mM Tris-HCl, 137 mM NaCl, 1 mM EDTA, 10% glycerol, 1% Triton-X 100, a protease inhibitor cocktail (Roche), pH 8.0) on ice. Ten to 30 μg of protein was subjected to SDS–polyacrylamide gel electrophoresis (SDS–PAGE) and transferred to a nitrocellulose membrane. Membranes were incubated with primary antibodies overnight at 4 °C, followed by incubations with secondary antibodies at RT for 1 h. Western blotting substrate plus (Pierce/Thermo Scientific) or Lumigen ECL Ultra (Lumigen) was used to detect protein bands according to the manufacturer’s instructions. NIH Image J software (version1.44p) or ImageQuant TL (GE) was used for densitometric analyses. Images are cropped for presentation. Uncropped images are presented in the Supplementary Material (Supplementary Figs 1-4).

### Immunoprecipitation

HEK293A cells were transfected for 48 h and lysed with lysis buffer (50 mM Tris-HCl, 137 mM NaCl, 1 mM EDTA, 10% glycerol, 1% Triton-X 100, a protease inhibitor cocktail, a phosphatase inhibitor cocktail (Roche), pH 8.0). Lysates were incubated for 10 min under rotation at 4 °C, followed by centrifugation at 15,000 g for 10 min at 4 °C. Lysates were precleared with 20 μl of agarose-coupled protein A (GE healthcare) and 1 μg of rat immunoglobulin G (Santa Cruz biochemistry). Precleared lysates were subjected to immunoprecipitation using 200 ng of the anti-HA antibody (clone 3F10, Roche) and 20 μl of agarose-coupled protein A at 4 °C for 2 h. The precipitate complexes were washed three times with lysis buffer. Immunoprecipitates were then analysed by immunoblotting.

For immunoprecipitation of endogenous Bcl2-L-13, cells were incubated with 20 nM bafilomycin A1 and DMSO or 5 μM CCCP for 1 h and collected with cold PBS, then sonicated (200 W, 90 s). After centrifugation at 700*g* for 5 min at 4 °C, lysates were precleared with 40 μl of agarose-coupled protein A and 1 μg of rabbit immunoglobulin G (Santa Cruz Biochemistry). Precleared lysates were subjected to immunoprecipitation using 1 μg of the anti-Bcl2-L-13 antibody and 40 μl of agarose-coupled protein A at 4 °C for 2 h. The precipitate complexes were washed three times with wash buffer (20 mM Tris-HCl, 5 mM NaCl, 2 mM EGTA, 0.1% Triton X-100, protease inhibitors, pH 7.4).

### GST pulldown

GST fusion protein of LC3B was induced in *Esherichia coli* strain BL21 (DE3) transfected with pGEX-LC3B by adding 1 mM isopropyl-β-D-thiogalactoside (Wako) for 3.5 h. Bacteria were lysed with PBS containing 1 mg ml^−1^ lysozyme (Wako), 30 U ml^−1^ Benzonase (Sigma), 1 mM dithiothreitol, 1 mM EDTA, 1% Triton X-100 and protease inhibitors. GST-LC3B was purified on Glutathione HiCap Matrix beads (Qiagen). For GST pulldown assay, HEK293A cells were transfected with expression constructs encoding protein of interest using Lipofectamine 2000, according to the manufacturer’s instructions. After 48 h, cells were lysed in lysis buffer (50 mM Tris-HCl, 137 mM NaCl, 1 mM EDTA, 10% glycerol, 1% Triton-X 100, a protease inhibitor cocktail, pH 8.0) and the lysates were incubated with 25 μg of GST-LC3B in 1 ml of wash buffer (50 mM Tris-HCl, 200 mM NaCl, 1 mM EDTA, 1% Igepal CA-630, 1 mM dithiothreitol, 10 mM MgCl_2_, protease inhibitors, pH 8.0) for 2 h at 4 °C and then washed five times with 1 ml of wash buffer. The precipitate complex was boiled with 2 × SDS–PAGE loading buffer for 3 min at 95 °C and analysed by SDS-PAGE. The lower half of gel was cut-off and stained with Coomassie Brilliant Blue (Wako) to visualize GST or GST-LC3B and upper half was transferred to a nitrocellulose membrane and immunoblotted with anti-HA antibody.

### Subcellular fractionation

Subcellular fractionation of HEK293A cells was carried out using a mitochondria isolation kit (Pierce/Thermo Scientific) according to the manufacturer’s instructions. For proteinase K digestion, mitochondria fraction was suspended in MB buffer (210 mM mannitol, 70 mM sucrose, 10 mM HEPES, 1 mM EDTA, pH 7.5) with 50 μg ml^−1^ proteinase K (Wako) and incubated for 30 min at RT. For analysis of membrane proteins, mitochondrial pellets were resuspended in 100 μl of MB buffer or MB buffer containing 0.1 M Na_2_CO_3_ (pH 11.5) and incubated on ice for 30 min^19^.

### Mitochondrial DNA quantification

DNA from HEK293A cells was extracted using the QIAamp DNA Mini Kit (Qiagen) according to the manufacturer’s instructions. The SyBR GreenER qPCR SuperMix (Invitrogen) was used for quantitative PCR with an ABI PRISM 7900HT sequence detection system (Applied Biosystems). The Human Mitochondrial DNA (mtDNA) Monitoring Primer Set (Takara) was used for amplification of mtDNA and nuclear DNA (nDNA), and data analysis was performed according to the manufacturer’s instructions.

### Statistical analysis

Results are shown as the mean±s.e.m. Paired data were evaluated by Student’s *t*-test. A one-way analysis of variance (ANOVA) followed by Tukey–Kramer’s *post hoc* test was used for multiple comparisons. A value of *P*<0.05 was considered statistically significant.

## Additional information

**How to cite this article:** Murakawa, T. *et al*. Bcl-2-like protein 13 is a mammalian Atg32 homologue that mediates mitophagy and mitochondrial fragmentation. *Nat. Commun.* 6:7527 doi: 10.1038/ncomms8527 (2015).

## Supplementary Material

Supplementary Information Supplementary Figures 1-4

## References

[b1] Westermann B. Mitochondrial fusion and fission in cell life and death. Nat. Rev. Mol. Cell. Biol. 11, 872–884 (2010). 21102612 10.1038/nrm3013

[b2] Mizushima N. & Komatsu M. Autophagy: renovation of cells and tissues. Cell 147, 728–741 (2011). 22078875 10.1016/j.cell.2011.10.026

[b3] Kim I., Rodriguez-Enriquez S. & Lemasters J. J. Selective degradation of mitochondria by mitophagy. Arch. Biochem. Biophys. 462, 245–253 (2007). 17475204 10.1016/j.abb.2007.03.034 PMC2756107

[b4] Mao K., Wang K., Liu X. & Klionsky D. J. The scaffold protein Atg11 recruits fission machinery to drive selective mitochondria degradation by autophagy. Dev. Cell. 26, 9–18 (2013). 23810512 10.1016/j.devcel.2013.05.024 PMC3720741

[b5] Twig G. . Fission and selective fusion govern mitochondrial segregation and elimination by autophagy. EMBO J. 27, 433–446 (2008). 18200046 10.1038/sj.emboj.7601963 PMC2234339

[b6] Okamoto K., Kondo-Okamoto N. & Ohsumi Y. Mitochondria-anchored receptor Atg32 mediates degradation of mitochondria via selective autophagy. Dev. Cell. 17, 87–97 (2009). 19619494 10.1016/j.devcel.2009.06.013

[b7] Kanki T., Wang K., Cao Y., Baba M. & Klionsky D. J. Atg32 is a mitochondrial protein that confers selectivity during mitophagy. Dev. Cell. 17, 98–109 (2009). 19619495 10.1016/j.devcel.2009.06.014 PMC2746076

[b8] Schweers R. L. . NIX is required for programmed mitochondrial clearance during reticulocyte maturation. Proc. Natl Acad. Sci. USA 104, 19500–19505 (2007). 18048346 10.1073/pnas.0708818104 PMC2148318

[b9] Liu L. . Mitochondrial outer-membrane protein FUNDC1 mediates hypoxia-induced mitophagy in mammalian cells. Nat. Cell. Biol. 14, 177–185 (2012). 22267086 10.1038/ncb2422

[b10] Youle R. J. & Narendra D. P. Mechanisms of mitophagy. Nat. Rev. Mol. Cell. Biol. 12, 9–14 (2011). 21179058 10.1038/nrm3028 PMC4780047

[b11] Huynh D. P., Dy M., Nguyen D., Kiehl T.-R. & Pulst S. M. Differential expression and tissue distribution of parkin isoforms during mouse development. Dev. Brain Res. 130, 173–181 (2001). 11675120 10.1016/s0165-3806(01)00234-6

[b12] Pawlyk A. C. . Novel monoclonal antibodies demonstrate biochemical variation of brain Parkin with age. J. Biol. Chem. 278, 48120–48128 (2003). 12972409 10.1074/jbc.M306889200

[b13] Matsuda N. . PINK1 stabilized by mitochondrial depolarization recruits Parkin to damaged mitochondria and activates latent Parkin for mitophagy. J. Cell Biol. 189, 211–221 (2010). 20404107 10.1083/jcb.200910140 PMC2856912

[b14] Goldberg M. S. . Parkin-deficient mice exhibit nigrostriatal deficits but not loss of dopaminergic neurons. J. Biol. Chem. 278, 43628–43635 (2003). 12930822 10.1074/jbc.M308947200

[b15] Kataoka T. . Bcl-rambo, a novel Bcl-2 homologue that induces apoptosis via its unique C-terminal extension. J. Biol. Chem. 276, 19548–19554 (2001). 11262395 10.1074/jbc.M010520200

[b16] Kirkin V. . A role for NBR1 in autophagosomal degradation of ubiquitinated substrates. Mol. Cell. 33, 505–516 (2009). 19250911 10.1016/j.molcel.2009.01.020

[b17] Narendra D., Tanaka A., Suen D. F. & Youle R. J. Parkin is recruited selectively to impaired mitochondria and promotes their autophagy. J. Cell Biol. 183, 795–803 (2008). 19029340 10.1083/jcb.200809125 PMC2592826

[b18] Fujiki Y., Hubbard A. L., Fowler S. & Lazarow P. B. Isolation of intracellular membranes by means of sodium carbonate treatment: application to endoplasmic reticulum. J. Cell Biol. 93, 97–102 (1982). 7068762 10.1083/jcb.93.1.97 PMC2112113

[b19] Zhao J. . The novel conserved mitochondrial inner-membrane protein MTGM regulates mitochondrial morphology and cell proliferation. J. Cell. Sci. 122, 2252–2262 (2009). 19535734 10.1242/jcs.038513

[b20] Chang C.-R. & Blackstone C. Dynamic regulation of mitochondrial fission through modification of the dynamin-related protein Drp1. Ann. NY Acad. Sci. 1201, 34–39 (2010). 20649536 10.1111/j.1749-6632.2010.05629.x PMC5585781

[b21] Kabeya Y. . LC3, a mammalian homologue of yeast Apg8p, is localized in autophagosome membranes after processing. EMBO J. 19, 5720–5728 (2000). 11060023 10.1093/emboj/19.21.5720 PMC305793

[b22] Katayama H., Kogure T., Mizushima N., Yoshimori T. & Miyawaki A. A sensitive and quantitative technique for detecting autophagic events based on lysosomal delivery. Chem. Biol. 18, 1042–1052 (2011). 21867919 10.1016/j.chembiol.2011.05.013

[b23] Kondo-Okamoto N. . Autophagy-related protein 32 acts as autophagic degron and directly initiates mitophagy. J. Biol. Chem. 287, 10631–10638 (2012). 22308029 10.1074/jbc.M111.299917 PMC3323008

[b24] Aoki Y. . Phosphorylation of serine 114 on Atg32 mediates mitophagy. Mol. Biol. Cell. 22, 3206–3217 (2011). 21757540 10.1091/mbc.E11-02-0145 PMC3164466

[b25] Farré J. C., Burkenroad A., Burnett S. F. & Subramani S. Phosphorylation of mitophagy and pexophagy receptors coordinates their interaction with Atg8 and Atg11. EMBO. Rep. 14, 441–449 (2013). 23559066 10.1038/embor.2013.40 PMC3642380

[b26] Hollville E., Carroll Richard G. & Cullen Sean P. Martin Seamus J. Bcl-2 family proteins participate in mitochondrial quality control by regulating Parkin/PINK1-dependent mitophagy. Mol. Cell. 55, 451–466 (2014). 24999239 10.1016/j.molcel.2014.06.001

[b27] Mizushima N., Yoshimori T. & Ohsumi Y. The role of Atg proteins in autophagosome formation. Ann. Rev. Cell Dev. Biol. 27, 107–132 (2011). 21801009 10.1146/annurev-cellbio-092910-154005

[b28] McLelland G. L., Soubannier V., Chen C. X., McBride H. M. & Fon E. A. Parkin and PINK1 function in a vesicular trafficking pathway regulating mitochondrial quality control. EMBO J. 33, 282–295 (2014). 24446486 10.1002/embj.201385902 PMC3989637

[b29] Dawson T. M., Ko H. S. & Dawson V. L. Genetic animal models of Parkinson's disease. Neuron 66, 646–661 (2010). 20547124 10.1016/j.neuron.2010.04.034 PMC2917798

[b30] Sandoval H. . Essential role for Nix in autophagic maturation of erythroid cells. Nature 454, 232–235 (2008). 18454133 10.1038/nature07006 PMC2570948

[b31] Frederick R. L., McCaffery J. M., Cunningham K. W., Okamoto K. & Shaw J. M. Yeast Miro GTPase, Gem1p, regulates mitochondrial morphology via a novel pathway. J. Cell Biol. 167, 87–98 (2004). 15479738 10.1083/jcb.200405100 PMC2172521

