# Supplementary material for: Bcl-2-like protein 13 is a mammalian Atg32 homologue that mediates mitophagy and mitochondrial fragmentation
Source: Nat Commun. 2015 Jul 6;6:7527. doi: 10.1038/ncomms8527 (PMC4501433; doi:10.1038/ncomms8527)

Supplementary Figure 1. Uncropped gel images from Figure 1.

Fig. 1b

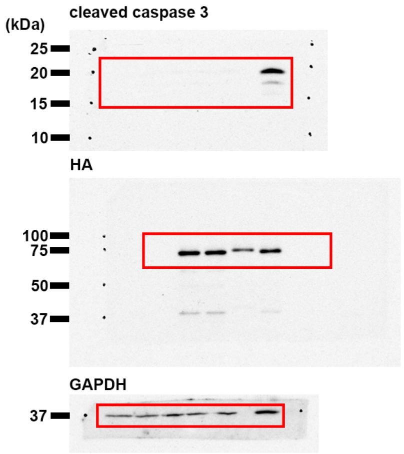

Fig. 1e

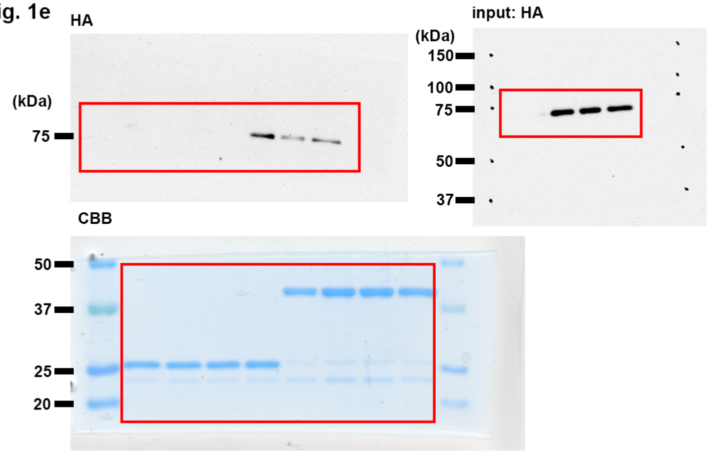

Fig. 1f

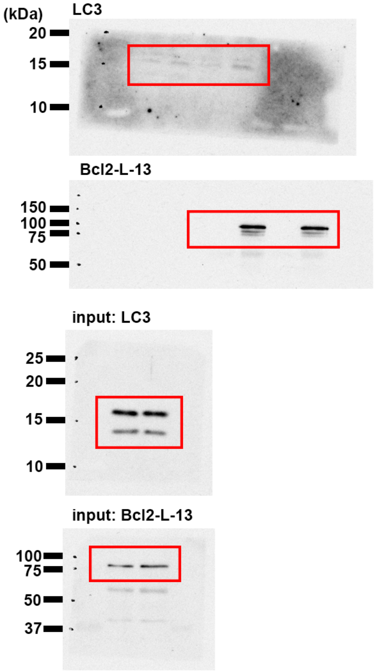

Fig. 1g

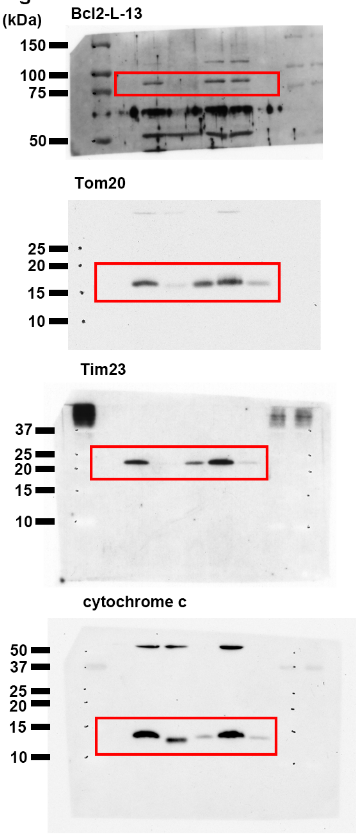

Fig. 1h

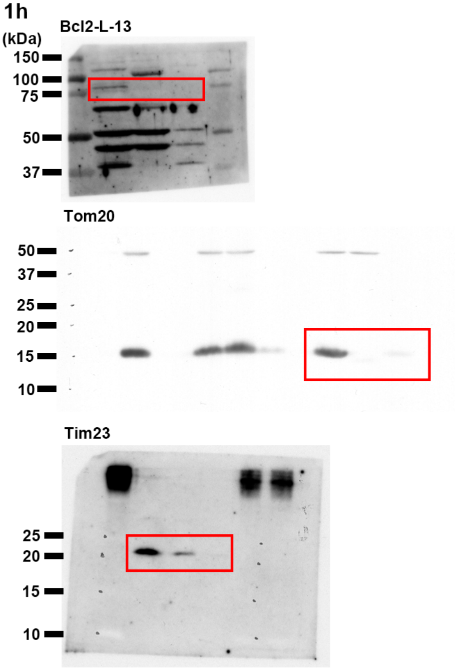

Supplementary Figure 2. Uncropped gel images from Figure 2 and Figure 4.

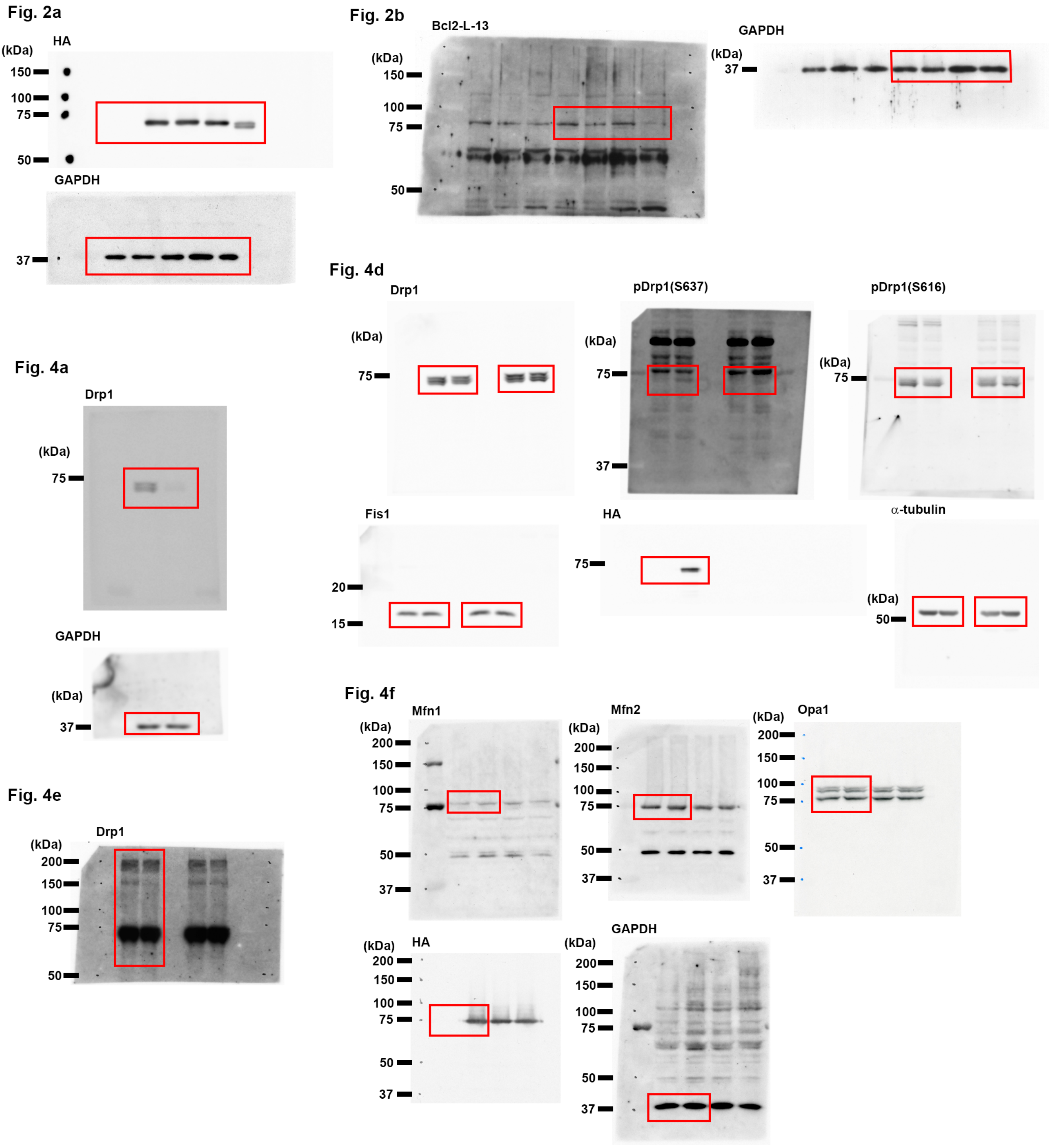

Supplementary Figure 3. Uncropped gel images from Figure 5 and Figure 7.

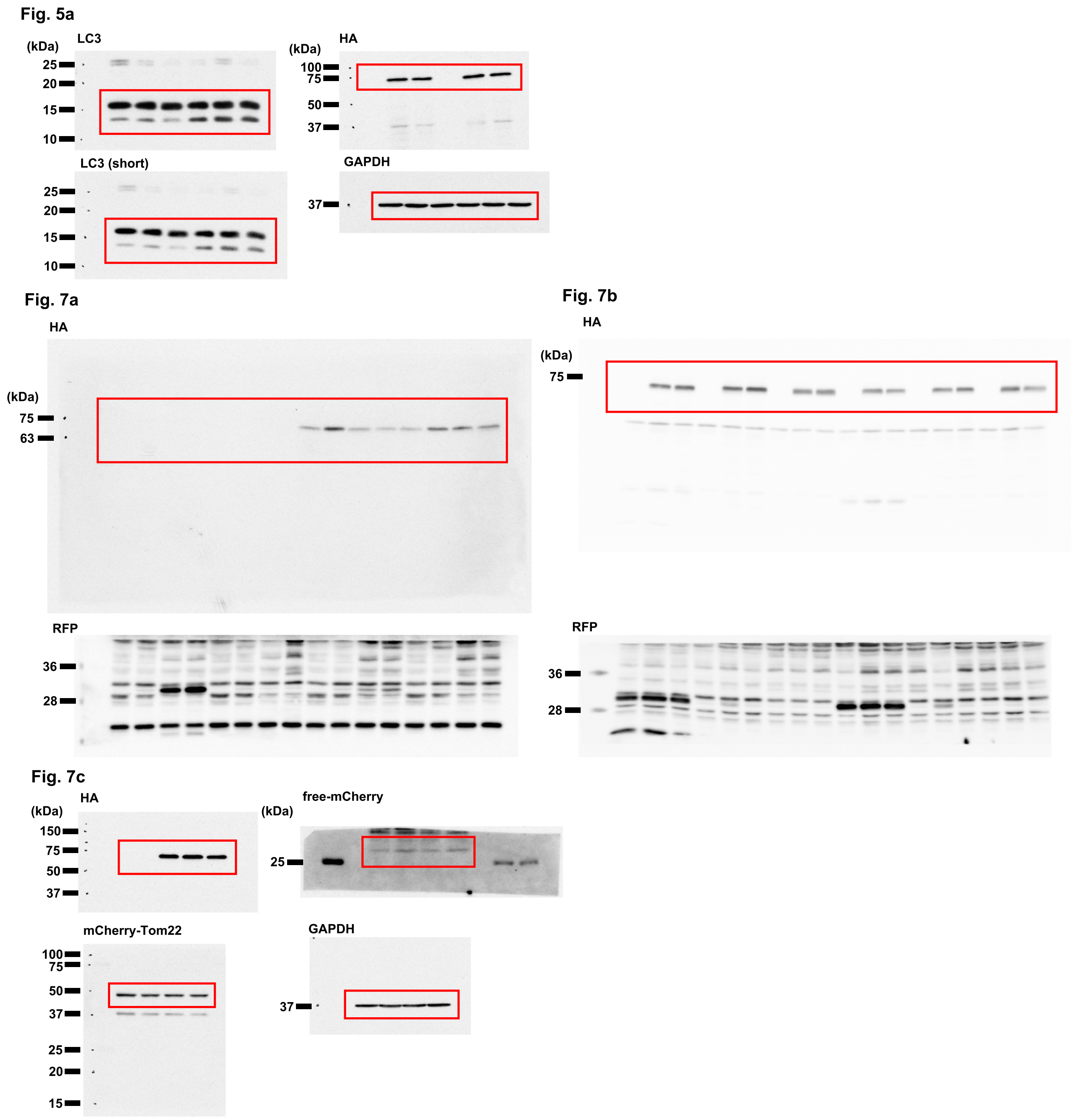

Supplementary Figure 4. Uncropped gel images from Figure 8.

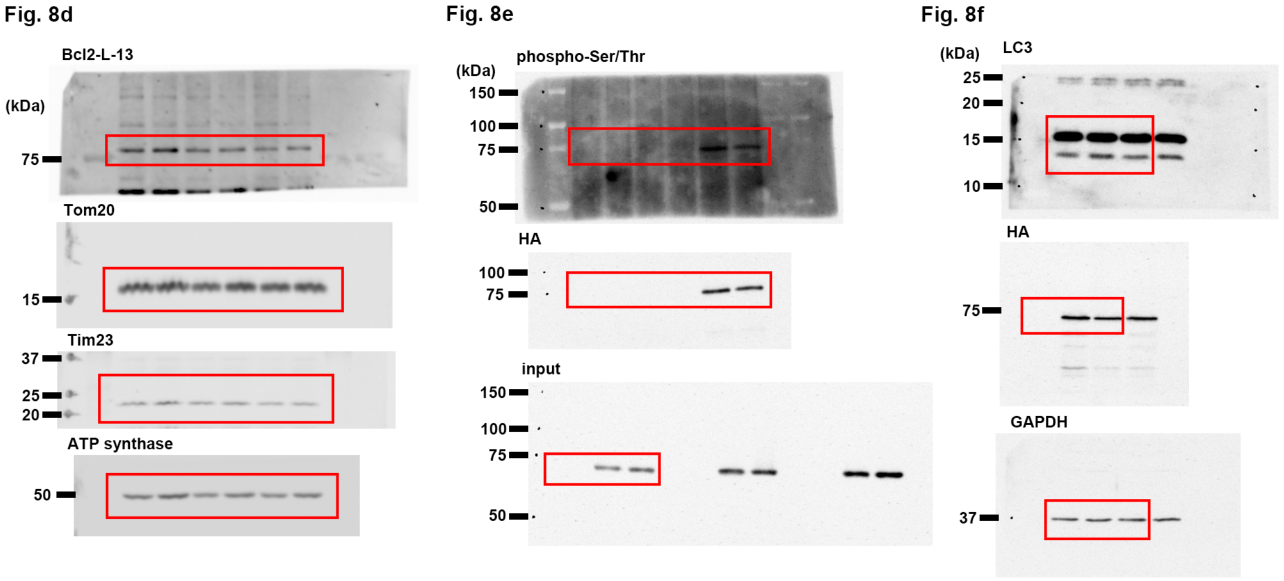

Supplement: Supplementary Information — Supplementary Figures 1-4 [file ncomms8527-s1.pdf]
